# Characteristics of hospitalised COVID-19 patients during the first two pandemic waves, Gauteng

**DOI:** 10.4102/sajid.v37i1.434

**Published:** 2022-09-30

**Authors:** Mpho L. Sikhosana, Waasila Jassat, Zinhle Makatini

**Affiliations:** 1 Department of Virology, Faculty of Health Sciences, University of the Witwatersrand, Johannesburg, South Africa; 2 Department of Public Health and Outbreak Response, National Institute of Communicable Diseases, Johannesburg, South Africa

**Keywords:** COVID-19, hospitalisation, in-hospital mortality, comorbidities

## Abstract

**Background:**

Gauteng province (GP) was one of the most affected provinces in the country during the first two pandemic waves in South Africa. We aimed to describe the characteristics of coronavirus disease 2019 (COVID-19) patients admitted in one of the largest quaternary hospitals in GP during the first two waves.

**Objectives:**

Study objectives were to determine factors associated with hospital admission during the second wave and to describe factors associated with in-hospital COVID-19 mortality.

**Method:**

Data from a national hospital-based surveillance system of COVID-19 hospitalisations were used. Multivariable logistic regression models were conducted to compare patients hospitalised during wave 1 and wave 2, and to determine factors associated with in-hospital mortality.

**Results:**

The case fatality ratio was the highest (39.95%) during wave 2. Factors associated with hospitalisation included age groups 40–59 years (adjusted odds ratio [aOR]: 2.14, 95% confidence interval [CI]: 1.08–4.27), 60–79 years (aOR: 2.49, 95% CI: 1.23–5.02) and ≥ 80 years (aOR: 3.39, 95% CI: 1.35–8.49). Factors associated with in–hospital mortality included age groups 60–79 years (aOR: 2.55, 95% CI: 1.11–5.84) and ≥ 80 years (aOR: 5.66, 95% CI: 2.12–15.08); male sex (aOR: 1.56, 95% CI: 1.22–1.99); presence of an underlying comorbidity (aOR: 1.76, 95% CI: 1.37–2.26), as well as being admitted during post–wave 2 (aOR: 2.42, 95% CI: 1.33–4.42).

**Conclusion:**

Compared to the recent omicron-driven pandemic waves characterised by lower admission rates and less disease severity among younger patients, COVID-19 in-hospital mortality during the earlier waves was associated with older age, being male and having an underlying comorbidity.

**Contribution:**

This study showed how an active surveillance system can contribute towards identifying changes in disease trends.

## Introduction

Five coronavirus disease 2019 (COVID-19) waves of infection have occurred in South Africa since the first case was reported in South Africa on 05 March 2020, with each wave driven by a predominant variant – the ancestral strain with an Asp614Gly mutation (first wave), the beta variant (second wave), the delta variant (third wave), the omicron B.1.1.529 variant (fourth wave) and the omicron BA.4 and BA.5 subvariants (fifth wave).^1,2^ By the end of the first year of the pandemic, 1 517 666 laboratory-confirmed cases and 50 462 deaths had been reported, and two waves of infections had occurred.^3,4^ Gauteng province (GP) was one of the worst affected provinces during the first year of the pandemic, with 405 860 cases (26.7%) and 9797 (19.4%) deaths reported.^3^ Of the province’s five districts, the Johannesburg district was the province’s COVID-19 epicentre.^5^ Charlotte Maxeke Johannesburg Academic Hospital (CMJAH), an academic quaternary hospital in the Johannesburg district of GP, was one of three healthcare facilities designated to manage COVID-19 patients in GP at the beginning of the pandemic. The aim of this study was to describe the characteristics of COVID-19 hospitalisations at CMJAH during the first two waves of the pandemic.

## Methods

In this retrospective review, the authors used secondary data from the South African national active hospital surveillance system, DATCOV, that had been used to capture national data for COVID-19 hospital admissions since 05 March 2020. At the time that the study was conducted, the DATCOV surveillance system collected data from hospitalised patients with a positive severe acute respiratory syndrome coronavirus 2 (SARS-CoV-2) reverse transcription polymerase chain reaction (RT-PCR) molecular test who were hospitalised for 1 full day or longer, irrespective of the reason for admission or age.^1^ The data were collected by clinicians and nurses at health-facility level. The data analysed for this study were only of patients admitted at CMJAH, a 1088-bed hospital in the City of Johannesburg in GP, with intensive and high-care facilities offering tertiary, secondary and highly specialised services.^5^ Demographic, clinical and outcome data, exposures such as occupation, as well as the comorbid diseases of confirmed SARS-CoV-2 cases that met the surveillance inclusion criteria were entered into the DATCOV surveillance system using a case report form based on a World Health Organization (WHO) COVID-19 case reporting tool.^6^ Individuals who were not classified as white, black, mixed race or Indian were reported as ‘other’ or ‘unknown’ in the surveillance system. For the purposes of this study, patients whose race was reported as ‘other’ or ‘unknown’ were reclassified as ‘race unknown’.

In order to compare this study’s findings with national data, the authors divided the study period into five wave periods using the methodology developed by the National Institute for Communicable Diseases, when the country’s weekly incidence risk was five admissions per 100 000 people at the beginning and at the end of the wave period.^4^ The wave periods were pre-wave 1 (05 March 2020 – 06 June 2020), wave 1 (07 June 2020 – 22 August 2020), post-wave 1 (23 August 2020 – 14 November 2020), wave 2 (15 November 2020 – 06 February 2021) and post-wave 2 (07 February 2021 – 27 March 2021).^6^ Case fatality risk per month was calculated by using the number of COVID-19 deaths for a particular month as the numerator and the sum of COVID-19 deaths and patients discharged alive for the same month as the denominator. Categorical data were summarised as frequencies and percentages, whereas numerical data were expressed as medians and interquartile ranges (IQRs). The chi-square test was used to compare proportions, and all *p* ≤ 0.05 were considered statistically significant.

Using two multivariable logistic regression models, the authors compared the characteristics of hospitalised COVID-19 patients admitted during the first and second waves and also determined the factors associated with in-hospital mortality. Factors included in the regression model were established risk factors based on previous studies. Factors used in the multivariable analysis comparing wave 1 and wave 2 were age, gender, comorbidity and race, and only data from these two wave periods were included in the model. Factors included in the multivariable analysis assessing risk factors for in-hospital mortality included age, gender, comorbidity, race and wave period, and data were included from all five wave periods. Analysis of in-hospital mortality was limited to patients whose outcome status was reported as either ‘COVID-related death’ or ‘discharged alive’. Patients who were either still in hospital, were transferred to another health facility or died of causes unrelated to COVID-19 were excluded. The race variable had more than 30% missing data, while complete or near-complete variables were age, gender and presence of comorbidity. The presence of comorbidity was reported as either yes or no. To account for missing data, the authors used multiple imputation to generate 100 imputed datasets that were used for further analyses. Factors with a significant *p* < 0.2 in the univariate analysis were included in the multivariate analysis, and only significant variables with a *p* < 0.05 were kept in the final model after manual backward elimination. Stata statistical software version 15 was used for data analysis (StataCorp, College Station, Texas, United States).

### Ethical considerations

Ethical approval was obtained from the Faculty of Health Sciences Research Ethics Committee of the University of the Witwatersrand (M210716).

## Results

There were 1861 SARS-CoV-2 patients admitted at CMJAH from 05 March 2020 to 27 March 2021 (Table 1), 964 (51.80%) of whom were female. There were more patients among the 40–49 years (336; 18.05%), 50–59 years (412; 22.14%) and 60–69 years (308; 16.55%) age groups. The median age (IQR) of all admitted cases was 50 years (IQR: 37–61). The median age was the highest in wave 2 (53; IQR: 40–63) and post-wave 2 (53; 42–63). The majority of the patients were black (1092; 58.68%) while the fewest patients admitted were of mixed race (23; 1.24%) or other race (35; 1.88%). Of the 1861 admitted patients, only 39 (2.10%) were healthcare workers (HCWs), 21 (53.85%) of whom were nurses, and 2 (5.13%) of whom were doctors. Higher proportions of HCWs were admitted during pre-wave 1 (8; 20.51%) and wave 1 (26; 66.67%).

**TABLE 1:** Characteristics of COVID-19 patients admitted at Charlotte Maxeke Hospital, 05 March 2020 – 27 March 2021.

Variable	Pre-wave 1 (05 March 2020 – 06 June 2020)				Wave 1 (07 June 2020 – 22 August 2020)				Post-wave 1 (23 August 2020 – 14 November 2020)				Wave 2 (15 November 2020 – 06 February 2021)				Post-wave 2 (07 February 2021 – 27 March 2021)				Total				*p*
	*n*	%	Median	IQR	*n*	%	Median	IQR	*n*	%	Median	IQR	*n*	%	Median	IQR	*n*	%	Median	IQR	*n*	%	Median	IQR	
**Age (years)**			42	32–54			49	37–60			44	31–61			53	40–63			53	42–63			50	37–61	< 0.001
0–9	2	1.32			20	2.11			3	2.44			7	1.33			0	0.00			32	1.72			-
10–19	3	1.97	-	-	20	2.11	-	-	4	3.25	-	-	4	0.76	-	-	1	0.91	-	-	32	1.72	-	-	-
20–29	18	11.84	-	-	81	8.54	-	-	17	13.82	-	-	35	6.63	-	-	8	7.27	-	-	159	8.54	-	-	-
30–39	48	31.58	-	-	165	17.41	-	-	29	23.58	-	-	79	14.96	-	-	15	13.64	-	-	336	18.05	-	-	-
40–49	31	20.39	-	-	192	20.25	-	-	18	14.63	-	-	99	18.75	-	-	20	18.18	-	-	360	19.34	-	-	-
50–59	23	15.13	-	-	210	22.15	-	-	18	14.63	-	-	130	24.62	-	-	31	28.18	-	-	412	22.14	-	-	-
60–69	22	14.47	-	-	146	15.40	-	-	21	17.07	-	-	99	18.75	-	-	20	18.18	-	-	308	16.55	-	-	-
70–79	2	1.32	-	-	90	9.49	-	-	10	8.13	-	-	56	10.61	-	-	11	10.00	-	-	169	9.08	-	-	-
80 +	3	1.97	-	-	22	2.32	-	-	3	2.44	-	-	19	3.60	-	-	4	3.64	-	-	51	2.74	-	-	-
Unknown	0	0.00	-	-	2	0.21	-	-	0	0.00	-	-	0	0.00	-	-	0	0.00	-	-	2	0.11	-	-	-
**Gender**																									0.054
Female	65	42.76	-	-	488	51.48	-	-	54	43.90	-	-	289	54.73	-	-	68	61.82	-	-	984	51.80	-	-	-
Male	87	57.24	-	-	459	48.42	-	-	69	56.10	-	-	239	45.27	-	-	42	38.18	-	-	896	48.15	-	-	-
**Race**																									< 0.001
Black African patients	97	63.82	-	-	469	49.47	-	-	53	43.09	-	-	380	71.97	-	-	93	84.55	-	-	1092	58.68	-	-	-
White patients	5	3.29	-	-	30	3.16	-	-	2	1.63	-	-	24	4.55	-	-	7	6.36	-	-	68	3.65	-	-	-
Indian patients	8	5.26	-	-	31	3.27	-	-	3	2.44	-	-	8	1.52	-	-	0	0.00	-	-	50	2.69	-	-	-
Other	0	0.00	-	-	5	0.53	-	-	2	1.63	-	-	21	3.98	-	-	7	6.36	-	-	35	1.88	-	-	-
Mixed race patients	4	2.63	-	-	8	0.84	-	-	0	0.00	-	-	9	1.70	-	-	2	1.82	-	-	23	1.24	-	-	-
Unknown	38	25.00	-	-	405	42.72	-	-	63	51.22	-	-	86	16.29	-	-	1	0.91	-	-	593	31.86	-	-	-
**Health worker**																									0.002
No	144	94.74	-	-	922	97.26	-	-	123	100.00	-	-	524	99.24	-	-	109	99.09	-	-	1822	97.90	-	-	-
Yes	8	5.26	-	-	26	2.74	-	-	0	0.00	-	-	4	0.76	-	-	1	0.91	-	-	39	2.10	-	-	-
**Health worker speciality**																									0.012
Nurse	4	50.00	-	-	15	57.69	-	-	0	0.00	-	-	1	25.00	-	-	1	100	-	-	21	53.85	-	-	-
Doctor	0	0.00	-	-	1	3.85	-	-	0	0.00	-	-	1	25.00	-	-	0	0.00	-	-	2	5.13	-	-	-
Allied health	0	0.00	-	-	2	7.69	-	-	0	0.00	-	-	0	0.00	-	-	0	0.00	-	-	2	5.13	-	-	-
Administrators or porters	0	0.00	-	-	0	0.00	-	-	0	0.00	-	-	2	50.00	-	-	0	0.00	-	-	2	5.13	-	-	-
Other	4	50.00	-	-	8	30.77	-	-	0	0.00	-	-	0	0.00	-	-	0	0.00	-	-	12	30.77	-	-	-
**Ward at admission**																									< 0.001
General ward	134	88.16	-	-	907	95.68	-	-	114	92.68	-	-	457	86.55	-	-	100	90.91	-	-	1712	91.99	-	-	-
Intensive care	16	10.53	-	-	21	2.22	-	-	8	6.50	-	-	55	10.42	-	-	9	8.18	-	-	109	5.86	-	-	-
High care	1	0.66	-	-	20	2.11	-	-	1	0.81	-	-	16	3.03	-	-	1	0.91	-	-	39	2.10	-	-	-
Isolation	1	0.66	-	-	0	0	-	-	0	0.00	-	-	0	0.00	-	-	0	0.00	-	-	1	0.05	-	-	-
**Highest level of care**																									< 0.001
General ward	133	87.50	-	-	881	92.93	-	-	111	90.24	-	-	415	78.60	-	-	89	80.91	-	-	1629	87.53	-	-	-
Intensive care	18	11.84	-	-	49	5.17	-	-	12	9.76	-	-	98	18.56	-	-	20	18.18	-	-	197	10.59	-	-	-
High care	1	0.66	-	-	18	1.90	-	-	0	0.00	-	-	15	2.84	-	-	1	0.91	-	-	35	1.88	-	-	-
Length of hospital stay in days	-	-	7	3–13	6 (3–10)		-	-	-	-	8	5–13	-	-	7	4–12	-	-	8	5–12	-	-	7	4–11	-
**Outcome**																									< 0.001
Discharged alive	131	86.18	-	-	744	78.48	-	-	104	84.55	-	-	368	69.70	-	-	84	76.36	-	-	1431	76.89	-	-	-
Died (COVID-19)	17	11.18	-	-	190	20.04	-	-	15	12.20	-	-	147	27.84	-	-	23	20.91	-	-	392	21.06	-	-	-
Transfer to other facility	3	1.97	-	-	7	0.74	-	-	0	0.00	-	-	10	1.89	-	-	3	2.73	-	-	23	1.24	-	-	-
Died (non-COVID-19)	1	0.66	-	-	6	0.63	-	-	4	3.25	-	-	3	0.57	-	-	0	0.00	-	-	14	0.75	-	-	-
In hospital	0	0.00	-	-	1	0.11	-	-	0	0.00	-	-	0	0.00	-	-	0	0.00	-	-	1	0.05	-	-	-

On admission, 1712 (91.99%) patients were admitted to a general ward and 109 (5.86%) to the intensive care unit (ICU). During their hospitalisation, some of the patients initially treated in the general ward required higher levels of care, such that by the end of the study period, 1629 (87.53%) patients were treated in the general wards, 197 (10.59%) in the ICU and 35 (1.88%) in high-care units. A higher proportion of patients admitted in the ICU were treated during wave 2 (98; 18.56%) and post-wave 2 (20; 18.18%). Over two-thirds of the patients (1431; 76.89%) were discharged alive, while 392 (21.06%) died due to COVID-19 complications. Because of the high proportion of missing data, the authors could not analyse the signs and symptoms with which the patients presented on admission. However, patients were classified according to reasons for admission, including ‘COVID-19 symptoms’ (1367 cases [85.87%]), ‘isolation’ (six cases [0.38%]) or admission for other reason (219 cases [13.76%]).

Regarding the clinical management of patients, frequently used medications included antibiotics, steroids and anticoagulants (Online Appendix 1, Table 1). There were 857 (32.12%) patients who received antibiotics, the use of which consistently decreased with each wave period. Over 700 patients (785, 29.42%) received steroids, with the proportion being roughly similar across each wave period. Two hundred and eighty (10.49%) patients received anticoagulants, the use of which increased in the latter part of the study period. There was low use of antivirals and immunotherapies; however, colchicine use continued during every wave period, with a total of 497 (18.63%) patients having been treated with this drug.

The monthly case fatality ratio (CFR) peaked at 18.98% in July 2020 during the first wave, while the peak monthly CFR in the second wave was 23.45% in December 2020 (Online Appendix 1, Table 2). Most COVID-19 associated deaths occurred in wave 1 (190) and wave 2 (147), with the mortality rates of 25.54% and 39.95%, respectively (Table 2).

**TABLE 2:** COVID-19 in-hospital case fatality ratio by wave period at Charlotte Maxeke Hospital, March 2020 – March 2021.

Outcome	Pre-wave 1 (05 March 2020 – 06 June 2020)	Wave 1 (07 June 2020 – 22 August 2020)	Post-wave 1 (23 August 2020 – 14 November 2020)	Wave 2 (15 November 2020 – 06 February 2021)	Post-wave 2 (07 February 2021 – 27 March 2021)	Total
Alive (*n*)	131	744	104	368	84	1431
Dead (*n*)	17	190	15	147	23	392
Mortality (%)	11.49	20.34	12.61	28.54	21.50	21.50

The most prevalent comorbidities among the patients were hypertension (567; 30.47%), diabetes (402; 21.60%) and human immunodeficiency virus (HIV) (204; 10.96%) (Online Appendix 1, Table 3). There were 36 patients (1.93%) who were admitted with active tuberculosis (TB), while 24 patients (1.29%) had a history of previous TB infection (Table 4). Of the 204 patients with HIV, information on combination antiretroviral (cART) use was available for 189 patients, of whom 143 (75.66%) were on cART, 28 (14.81%) were treatment naïve and information on cART use was not known in 18 (9.52%).

**TABLE 3:** Univariate and multivariate analyses of factors associated with admission to hospital in the first and second COVID-19 waves at Charlotte Maxeke Hospital, 05 March 2020 – 27 March 2021.

Variable	Wave period				Univariate		Multivariate	
	Cases in wave 1		Cases in wave 2		OR	95% CI	OR	95% CI
	*n*	%	*n*	%				
**Age (years) (*n*= 1474)**								
0–19	40	4.23	11	2.08	1 (ref)	-	1 (ref)	-
20–39	246	26.00	114	21.59	**1.68**	**0.83–3.40**	1.71	0.85–3.47
40–59	402	42.49	229	43.37	**2.07**	**1.04–4.11**	**2.14**	**1.08–4.27**
60–79	236	24.95	155	29.36	**2.39**	**1.19–4.80**	**2.49**	**1.23–5.02**
≥ 80	22	2.33	19	3.60	**3.14**	**1.27–7.78**	**3.39**	**1.35–8.49**
**Gender (*n* = 1475)**								
Female	488	51.53	289	54.73	1 (ref)	-	-	-
Male	459	48.47	239	45.27	0.88	0.71–1.09	-	-
**Comorbidities (*n* = 1476)**								
No	427	45.04	231	43.75	1 (ref)	-	-	-
Yes	521	54.96	297	56.25	1.05	0.85–1.31	-	-
**Race (*n* = 959)**								
White patients	30	5.58	24	5.70	1 (ref)	-	1 (ref)	-
Mixed race patients	8	1.49	9	2.14	1.27	0.47–3.42	1.26	0.47–3.44
Indian patients	31	5.76	8	1.90	**0.30**	**0.14–0.67**	**0.29**	**0.13–0.64**
Black patients	469	87.17	380	90.26	0.99	0.57–1.71	0.92	0.53–1.59

Note: Statistically significant results in bold.

OR, odds ratio; CI, confidence interval; ref, reference.

Factors associated with being hospitalised during the second wave included age groups 40–59 years (adjusted odds ratio [aOR]: 2.14, 95% confidence interval [CI]: 1.08–4.27), 60–79 years (aOR: 2.49, 95% CI: 1.23–5.02) and ≥ 80 years (aOR: 3.39, 95% CI: 1.35–8.49) compared to the reference age group 0–19 years (Table 3). Compared to being white, being Indian was a factor less common during the second wave (aOR: 0.29, 0.13–0.64).

Factors associated with COVID-19 in-hospital mortality included age groups 60–79 years (aOR: 2.55, 95% CI: 1.11–5.84) and ≥ 80 years (aOR: 5.66, 95% CI: 2.12–15.08) compared to the age group 0–19 years; male gender (aOR: 1.56, 95% CI: 1.22–1.99); having a comorbid condition including hypertension, diabetes and HIV (aOR: 1.76, 95% CI: 1.37–2.26), as well as being admitted during post-wave 2 compared to the first wave (aOR: 2.42, 95% CI: 1.33–4.42) (Table 4).

**TABLE 4:** Univariate and multivariate analyses of factors associated with mortality due to COVID-19 at Charlotte Maxeke Hospital, 05 March 2020 – 27 March 2021.

Variable	Outcome				Univariate		Multivariate	
	Discharged alive		Died		OR	95% CI	OR	95% CI
	*n*	%	*n*	%				
**Age (year) (*n* = 1821)**								
0–19	57	80.06	7	10.94	1 (ref)	-	1 (ref)	-
20–39	437	90.48	46	9.52	0.86	0.37–1.99	0.74	0.31–1.73
40–59	594	78.36	164	21.64	**2.25**	**1.01–5.02**	1.52	0.67–3.46
60–79	316	67.96	149	32.04	**3.84**	**1.71–8.62**	**2.55**	**1.11–5.84**
> 80	26	50.98	25	49.02	**7.83**	**3.00–20.41**	**5.66**	**2.12–15.08**
**Gender (*n* = 1822)**								
Female	770	81.31	177	18.69	1 (ref)	-	-	-
Male	660	75.43	215	24.57	**1.42**	**1.13–1.77**	**1.56**	**1.22–1.99**
**Comorbidities (*n* = 1823)**								
No	742	85.09	130	14.91	1 (ref)	-	-	-
Yes	689	72.45	262	27.55	**2.17**	**1.72–2.74**	**1.76**	**1.37–2.26**
**Race (*n* = 1202)**								
White patients	43	64.18	24	35.82	1 (ref)	-	1 (ref)	-
Mixed race patients	19	82.61	4	17.39	0.80	0.27–2.40	0.69	0.22–2.16
Indian patients	38	77.55	11	22.45	1.00	0.50–2.00	0.84	0.39–1.80
Black patients	831	78.17	232	21.83	**2.02**	**1.21–3.37**	1.67	0.97–2.89
**Wave period (*n* = 1823)**								
Pre-wave 1	131	88.51	17	11.49	1 (ref)	-	1 (ref)	-
Wave 1	744	79.66	190	20.34	1.90	0.93–3.87	1.78	0.85–3.75
Post-wave 1	104	87.39	15	12.61	0.90	0.43–1.89	0.97	0.45–2.09
Wave 2	368	71.46	147	28.54	**1.77**	**1.01–3.11**	1.62	0.90–2.91
Post-wave 2	84	78.50	23	21.50	**2.77**	**1.55–4.92**	**2.42**	**1.33–4.42**

Note: Statistically significant results in bold.

OR, odds ratio; CI, confidence interval; ref, reference.

## Discussion

This study showed that being ≥ 40 years was associated with COVID-19 hospitalisation during the second wave, while COVID-19 in-hospital mortality was associated with being ≥ 60 years, being male and having an underlying comorbid condition. These findings have been described during the early waves of the pandemic in the South African setting at the national level^7^ as well as in systematic reviews.^8,9,10,11,12^ However, as the pandemic has progressed, with the recent waves of infection being driven by the less-severe omicron variants, it has been characterised by lower admission rates, shorter length of hospital stays and reduced disease severity.^1,13,14^ The trend of higher admission rates among younger compared to older people during the more recent pandemic waves has been attributed to immunity induced by previous SARS-CoV-2 infection, vaccination or hybrid immunity.^13,14^ What has also been characteristic of the omicron-driven wave is the decoupling of COVID-19 hospital admissions and deaths, and this has also been attributed to the background population immunity.^14^

This study’s finding that ages ≥ 60 years were significantly associated with in-hospital mortality was consistent with what has been reported both locally and in other settings during the earlier pandemic waves.^6,15,16,17^ Increasing age is associated with an increase in prevalence of comorbidities, and this could explain the increased risk of COVID-19 mortality among older patients. Although there has been an overall reduction in the number of recorded deaths across all age groups older than 17 years during the fourth wave in South Africa compared to earlier waves,^14^ proportions of in-hospital deaths have remained the highest among patients ≥ 60 years.^1^

Being male has been identified as a risk factor for COVID-19-related adverse clinical outcomes, even during the recent less severe omicron-driven wave.^1,7,18^ This finding may be explained by biological gender-specific differences in immune responses and health-seeking behaviours.^19,20,21^ Male predominance has also been described in the setting of epidemics associated with two other coronaviruses, that is, severe acute respiratory syndrome coronavirus 1 (SARS CoV-1, 2002–2003) and Middle East Respiratory Syndrome (MERS, 2012–2013).^18^

The association between the presence of an underlying comorbid condition and adverse clinical outcomes has been reported in the literature locally and globally in both adults and children throughout the pandemic, including during the recent less-severe waves.^1,7,22,23^ Comorbidities associated with both hospitalisation and mortality included hypertension, diabetes and HIV, and this is in keeping with the background prevalence of these conditions in South Africa.^24^

The monthly CFR at CMJAH peaked in July 2020 during the first wave, which is similar to the peak reported from national data, while the peak monthly CFR during the second wave was in December 2020.^7^ This study also revealed that mortality during wave 2 was higher compared to mortality during wave 1. This increased in-hospital mortality during wave 2 compared to wave 1 has been associated with higher admission rates of older patients, higher admissions in the public healthcare sector and the possible contribution of the more transmissible circulating beta virus variant.^6^ However, as previously mentioned, compared to the preceding three waves, the recent omicron-driven wave has been characterised by reduced disease severity and lower case-fatality ratios.^1^

The management of patients with COVID-19 has evolved since the emergence of the disease.^25,26,27,28^ The use of steroids throughout the study period was not in keeping with current guidelines that have highlighted the lack of benefit of using steroids in patients in whom oxygen support was not required. Similarly, the continued use of antibiotics throughout the study period was also inconsistent with clinical management recommendations, because empirical antibiotic use has since been discouraged owing to the uncommon reporting of bacterial co-infections in COVID-19 patients in this study and other settings.^28,29,30,31,32^ Evidence of the occurrence of thrombotic events among COVID-19 patients was available as early as April 2020.^33,34,35,36^ By the end of the first wave, there was significant evidence linking COVID-19 and the incidence of venous thromboembolism (VTE), necessitating the inclusion of VTE prophylaxis in COVID-19 in-patient treatment guidelines.^29^ This would explain the increase in use of anticoagulants from post-wave 1 in the current study population.

In this study, HCWs were more likely to be hospitalised during pre-wave 1 (8; 5.26%), with the proportion of hospitalised HCW cases decreasing as the pandemic progressed. This was most likely due to increased awareness of the disease and the enforced infection prevention control protocols as more information about COVID-19 became available.

The main strength of this study was the use of a large and comprehensive dataset that included a diverse patient population, allowing the identification of important factors associated with hospital admissions during the different pandemic waves, as well as in-hospital mortality. However, this study had several limitations. Firstly, any information bias that could have been introduced during the collection of data would affect the overall study findings. Also, patients’ clinical records were not reviewed; therefore, the authors could not conduct a validation check to identify errors that may have occurred during data collection. Lastly, the study is a retrospective single-centre study, and the findings may not be generalisable to other settings.

## Conclusion

Although this study found that older age, being male and having an existing comorbidity were factors associated with COVID-19 in-hospital mortality, the pandemic in South Africa has since changed. Lower admission rates and reduced disease severity have characterised the latter omicron-driven waves. This reflects the importance of having efficient hospital as well as phylogenetic surveillance systems that allow for the identification of shifts in disease trends and the genetic evolution of the associated pathogen.
